# Instruments for Short‐Term (24 h) Violence Risk Assessment and Strategies for Managing Violence Risk Among Adolescents With Risk for Violent Behaviour: A Systematic Review

**DOI:** 10.1111/inm.70110

**Published:** 2025-08-04

**Authors:** Laura Väätäinen, Maiju Björkqvist, Yan Li, Veikko Pelto‐Piri, António Ferreira, Tella Lantta

**Affiliations:** ^1^ Department of Nursing Science, Faculty of Medicine University of Turku Turku Finland; ^2^ School of Nursing Hong Kong Polytechnic University Hong Kong SAR China; ^3^ University Health Care Research Center Faculty of Medicine and Health, Örebro University Örebro Sweden; ^4^ Centro Hospitalar Universitario De Lisboa Central (CHULC) Lisbon Portugal; ^5^ Centre for Forensic Behavioural Science at Swinburne University of Technology Melbourne Victoria Australia; ^6^ Department of Biomedical, Metabolic and Neuroscience University of Modena and Reggio Emilia Reggio Emilia Italy

**Keywords:** adolescent, mental health, outcomes, risk assessment, risk management, violence

## Abstract

Short‐term (24 h) violence risk assessment and management can reduce violence in institutional settings, yet they remain understudied in adolescent populations. This systematic review aimed to identify instruments used for short‐term violence risk assessment and strategies for managing violence risk among adolescents in institutional settings, as well as to evaluate related outcomes. PRISMA was used as an evidence‐based minimum set of items for reporting systematic reviews. The literature search (March 2024 and March 2025) was conducted in PubMed, PsycINFO, Web of Science, CINAHL, The Cochrane Library and Scopus, and references from selected studies were reviewed. Data extraction and analysis were performed in Covidence. Nine studies met inclusion criteria describing six assessment instruments: DASA, DASA‐YV, V‐RISK‐Y, Kennedy Axis V, Pedi‐BEWS and BVC. No studies regarding strategies for short‐term violence risk management were identified. DASA‐YV, BVC and V‐RISK‐Y predicted violence among adolescents within 24 h (AUC = 0.70–0.95); DASA predicted violence moderately (AUC = 0.50–0.69). Pedi‐BEWS (ICC = 0.83) and Kennedy Axis V (ICC = 0.79) demonstrated similar inter‐rater reliability. Due to the lack of studies, firm conclusions on the best instrument for clinical practice in institutional settings remained elusive. Further research is necessary to ascertain if youth‐specific instruments (e.g., DASA‐YV, V‐RISK‐Y) predict violence more effectively than non‐age‐specific instruments (e.g., DASA). The lack of youth engagement in violence risk assessment stands out clearly. Scoring was done by the staff, mostly by nurses. Future studies should involve adolescents in the scoring or evaluation of assessment and management. There is a need for evidence‐based recommendations for youth engagement.

## 1 Introduction

Adolescence is a time of significant change and vulnerability, with an increase in violent expression and other risky behaviours (David‐Ferdon et al. 2021). Violence may be a symptom of, for example, mental health issues (Ruchkin et al. 2023), bullying, experiences of violence, isolation and various individual or family attributes or conditions (Staus 2024). Substance use is often linked to violent outcomes (Zhong et al. 2020). For a young person who uses violence, the consequences can be far‐reaching. The use of violence at a young age also increases the risk of violence later in life as an adult, as well as the possibility of becoming a victim. In addition, violence is a risk factor for many physical, psychological and social problems (Selekman et al. 2023).

Youth violence occurs at every level of society, including care facilities that offer round‐the‐clock care and support, for example, psychiatric inpatient care, emergency departments and community mental health settings (O'Rourke et al. 2018). These environments, for example, could be locked units where an individual's freedom of movement and contact with other individuals is restricted. Such restrictions are usually justified for reasons of safety, growth and development. In these environments, staff are most likely to be the victims of violence, followed by peers in the facilities and family members of the youths (Joyes et al. 2021). Mental health nurses are frequently the first responders to violence and are pivotal in both assessing and managing risk in inpatient settings (Simpson and McKenna 2014). For example, violent incidents recorded in Greece averaged 10.46 incidents per week or 1.49 incidents per day in the acute adolescent psychiatric units (Panagiotou et al. 2019). Violent incidents have both direct and indirect costs for society, which include an increase in staff sick leave (Andersen and Christensen 2022) and an impact on the physical, social and emotional health of both the youth and their families (David‐Ferdon et al. 2021). In addition to the negative consequences of youth violence both for the perpetrators and those who are exposed to violence, there are significant economic costs (Irwing‐Rogers et al. 2020). This means that there are multiple reasons to make efforts to create safe care environments by preventing and reducing violence in institutional settings (Dike et al., 2021).

Violence risk management is defined as any psychosocial or nursing practices aiming to lower the risk of violent behaviour in a care setting (Heilbrun et al. 2021). Identifying adolescents who require additional support and intervention to prevent or manage aggression relies on accurate risk assessment. Identifying adolescents likely to behave aggressively can help allocate existing resources more efficiently in institutional settings while reducing unnecessary restrictive measures (Chu et al. 2012). Risk assessment instruments enable professionals to manage and reduce violence risk and involve planning and implementing prevention strategies (Viljoen et al. 2018). Several valid violence risk assessment instruments can be used to assess the risk of aggression in institutional settings (Maguire et al. 2019). Based on a previous review, SAVRY (The Structured Assessment of Violence Risk in Youth; Borum et al. 2003), VRS‐YV (Violence Risk Scale—Youth Version; Lewis et al. 2004), START:AV (Short‐Term Assessment of Risk and Treatability: Adolescent Version; Viljoen et al. 2014) and DASA‐YV (Dynamic Assessment of Situational Aggression—Youth Version, Kasinathan et al. 2015) have been identified as suitable violence risk assessment instruments for young people (Koh et al. 2020). While there are numerous violence risk assessment instruments available for adults, there are far fewer specifically designed for youth.

Most previous reviews have centred on various aspects such as adult populations (Dickens et al. 2020), youth groups (Koh et al. 2020) and studies analysing the predictive validity of particular risk assessment tools like the Structured Assessment of Protective Factors (SAPROF; de Vogel et al. 2009), as well as targeted protective factors (Koh et al. 2022). Systematic reviews addressing strategies for managing violence risk in youth have predominantly examined non‐institutional settings, including outpatient facilities and schools (e.g., Haktanir et al. 2023), or therapeutic approaches that demand extensive training, such as Dialectical Behaviour Therapy (DBT; Linehan 1993). Nonetheless, there remains a lack of comprehensive data on short‐term (< 24 h) risk assessments, violence management strategies and the outcomes of these interventions for adolescents in institutional settings. Furthermore, evaluating the efficacy of risk assessments and management practices among this demographic is essential to ensure they function as intended.

In this review, we were particularly interested in discovering what violence risk assessment instruments and concrete management strategies have previously been studied in adolescents and the results they have achieved. This review aims to explore evidence‐based, youth‐appropriate, non‐invasive and non‐coercive approaches to managing the risk of violent behaviour that could be used together with adolescents. Detailed information on the requirements for their use is needed to support service‐level decision‐making regarding the suitability of the instruments for use in specific clinical settings.

## 2 Aim

This systematic review was conducted to synthesise the available evidence on instruments used for short‐term violence risk assessment and concrete strategies for the short‐term management of violence risk in adolescents in an institutional setting. The following research questions guided our review:

1. Which instruments are used for short‐term violence risk assessment among adolescents with a risk for violent behaviour in institutional settings?

2. Which strategies are used for short‐term violence risk management among adolescents with a risk for violent behaviour in institutional settings?

3. What kind of outcomes have been achieved with these instruments and strategies for violence risk assessment and management among adolescents in institutional settings?

## 3 Methods

This systematic review was guided by the PRISMA (the Preferred Reporting Items for Systematic Reviews and Meta‐Analyses) reporting protocol (Page et al. 2021) and was registered in the PROSPERO database (CRD42024505940).

### 3.1 Eligibility Criteria

In this systematic review, violence was defined as the use of direct physical actions to harm other people (peers or caregivers) or objects and verbal threats against other people (David‐Ferdon et al. 2016). Violence risk was defined as the likelihood that a violent incident will occur in the impending 24 h (Dickens et al. 2020). Short‐term violence risk assessment is defined as an instrument whose goal is to assess and manage the risk of violent behaviour in the following 24 h (Ogloff and Daffern 2006). Structured assessment of short‐term violence risk is based on identified violence risk factors and their capability to predict future violent behaviour (National Collaborating Centre for Mental Health (UK) 2015).

In this systematic review, a PICO method was used to identify the criteria to determine which research studies will be included as follows: population (adolescents), interventions (assessment of short‐term violence risk instruments and management strategies), constant variable (risk of violent behaviour) and outcomes in institutional settings. Initially, studies that had the majority (> 50%) of adolescents aged between 13 and 17 years were included. However, most of the research articles did not report this proportion of adolescents (in joint sampling that usually included children and adolescents), so the criterion was broadened. Ultimately, studies that mentioned adolescents (age range varied from 4 to 21 years) were incorporated into the study. It should be mentioned that adolescents can also be in adult departments. As a type of intervention, short‐term (24 h) violence risk instruments based on actuarial and structured clinical judgement (SCJ) (Doyle and Dolan 2002) were included. The study also considered: the type of participants and institutional settings, inpatient care units, civil or forensic psychiatric care units, foster care centres (including substance or alcohol abuse) and prison/juvenile offender populations. There were no restrictions made as regards the health or care status or diagnostic restrictions of the participants. Studies reporting sexual, domestic, self‐harm and family violence were excluded from this systematic review as the focus was on short‐term violence risk toward others in institutional settings. The study was excluded if it was conducted in outpatient settings or long‐term care settings primarily for learning disabilities, intellectual disabilities and autism. Study types were not restricted for comparison, as the study was not focused solely on randomised controlled trials. In addition, empirical studies representing qualitative, quantitative, or mixed‐methods designs published in peer‐reviewed journals were also included in this systematic review. For those papers that met the criteria but were not available in full text, the authors were contacted before the article was rejected.

### 3.2 Search Strategy

The search was conducted in February 2024 and re‐run in March 2025 as a systematic search in the databases PubMed, PsycINFO, Web of Science, CINAHL, The Cochrane Library and Scopus. The database search was complemented by a manual search to screen potential studies in reference lists of the eligible studies and review articles. In developing the search strategy, an information specialist was consulted, and search terms were verified. The PICO method was used to define keywords to describe the desired population (adolescents), constant variable (risk of violent behaviour), interventions (assessment of short‐term violence risk and management) and outcomes in institutional settings. The terms were supplemented on a database‐by‐database basis by adding, for example, MESH terms in PubMed. An example of a used search strategy for CINAHL is presented in Table 1. There were no limitations applied to the language of publication to ensure all research conducted in our area of interest was identified and incorporated into the review. However, the date of publication was limited from 2000 to the present, as the first instrument for adolescents was published in the early 21st century.

**TABLE 1:** Search strategy terms.

Adolescents (P)	CINAHL	youth* OR ‘young people’ OR youngster* OR junior* OR juven* OR adoles* OR teen* OR minor*
	PubMed	(‘young people’[tw] OR youngster*[tw] OR junior*[tw] minor*[tw] OR youth[tw] OR juven*[tw] OR adolescent*[tw] OR teen*[tw] OR adoles*[tw] OR ‘adolescent’[Mesh] OR ‘Minors’[Mesh])
	PsycINFO	(youth* OR ‘young people’ OR youngster* OR junior* OR juven* OR adoles* OR teen* OR minor* OR DE ‘Youth Mental Health’)
	Cochrane Library	youth* OR young NEXT people OR youngster* OR junior* OR juven* OR adoles* OR teen* OR minor*
	Scopus	(youth* OR ‘young people’ OR youngster* OR junior* OR juven* OR adoles* OR teen* OR minor*)
	Web of science	(youth* OR ‘young people’ OR youngster* OR junior* OR juven* OR adoles* OR teen* OR minor*)
Problem (P)	CINAHL	anger OR rage OR violen* OR aggress* OR force* OR assault* OR agitation
	PubMed	(rage[tw] OR anger[tw] OR violen*[tw] OR aggress*[tw] OR force*[tw] OR assault*[tw] OR agitation[tw] OR violence*[tw] OR ‘aggression’[Mesh] OR ‘violence’[Mesh] OR ‘Rage’[Mesh])
	PsycINFO	(anger OR violen* OR aggress* OR force* OR assault* OR agitation OR DE ‘Violence’ OR DE ‘Patient Violence’ OR DE ‘Workplace Violence’ OR DE ‘Violence Prevention’ OR DE ‘Aggressive Behaviour’ OR DE ‘Attack Behaviour’ OR DE ‘Coercion’ OR DE ‘Conflict’ OR DE ‘Microaggression’ OR DE ‘Threat’)
	Cochrane Library	anger OR rage OR violen* OR aggress* OR force* OR assault* OR agitation
	Scopus	(anger OR violen* OR rage OR aggress* OR force* OR assault* OR agitation)
	Web of science	(anger OR rage OR violen* OR aggress* OR force* OR assault* OR agitation)
Short‐term (I)	CINAHL	imminent OR ‘short‐term’ OR ‘short‐range’ OR ‘24 h’ OR ‘24‐h period’ OR ‘24 h’ OR immediate OR instant OR ‘one day’ OR ‘day and night’ OR dynamic OR rapid OR prompt
	PubMed	(imminent[tw] OR ‘short‐term’[tw] OR ‘short‐range’[tw] OR ‘24 h’[tw] OR ‘24‐h period’[tw] OR ‘24 h’[tw] OR immediate[tw] OR instant[tw] OR ‘one day’[tw] OR ‘day and night’[tw] OR dynamic[tw] OR rapid[tw] OR prompt[tw])
	PsycINFO	(imminent OR ‘short‐term’ OR ‘short‐range’ OR ‘24 h’ OR ‘24‐h period’ OR ‘24 h’ OR immediate OR instant OR ‘one day’ OR ‘day and night’ OR dynamic OR rapid OR prompt)
	Cochrane Library	short NEXT term* OR immediate* OR instant* OR one NEXT day OR dynamic* OR rapid* OR prompt* OR 24 h* OR 24 NEXT hour* NEXT period OR 24 NEXT hour*
	Scopus	(imminent OR ‘short‐term’ OR ‘short‐range’ OR ‘24 h’ OR ‘24‐h period’ OR ‘24 h’ OR immediate OR instant OR ‘one day’ OR ‘day and night’ OR dynamic OR rapid OR prompt)
	Web of science	(imminent OR ‘short‐term’ OR ‘short‐range’ OR ‘24 h’ OR ‘24‐h period’ OR ‘24 h’ OR immediate OR instant OR ‘one day’ OR ‘day and night’ OR dynamic OR rapid OR prompt)
Interventions (I)	CINAHL	‘nonpharmacological intervention’ OR ‘noninvasive intervention’ OR ‘non‐pharmacological intervention’ OR ‘non‐invasive intervention’ OR managem* OR ‘behavioural intervention*’ OR ‘crisis intervention*’ OR ‘risk assessment*’ OR ‘assessment tool’ OR ‘assessment method’ OR manag* OR ‘risk management*’ OR ‘management tool’ OR ‘management method’ OR evaluat* OR ‘evaluation tool’ OR ‘evaluation method’ OR estimat* OR ‘estimation tool’ OR ‘estimation method’ OR evaluat* OR ‘evaluation tool’ OR ‘evaluation method’ OR apprais* OR ‘appraisal tool’ OR ‘appraisal method’ OR intervent* OR tool* OR ‘nursing intervention’ OR ‘psychosocial intervention’ OR ‘sensory intervention*’ OR ‘sensory regulation’
	PubMed	(‘nonpharmacological intervention’[tw] OR ‘non‐pharmacological intervention’[tw] OR ‘non‐invasive intervention’[tw] OR ‘noninvasive intervention’[tw] OR ‘sensory modulation’[tw] OR ‘behavioural intervention*’[tw] OR ‘crisis intervention*’[tw] OR ‘risk assessment*’[tw] OR ‘assessment tool’ [tw] OR ‘assessment method’[tw] OR management*[tw] OR ‘risk management*’[tw] OR ‘management tool’ [tw] OR ‘management method’ [tw] OR evaluat*[tw] OR ‘evaluation tool’ [tw] OR ‘evaluation method’[tw] OR estimat*[tw] OR ‘estimation tool’ [tw] OR ‘estimation method’[tw] OR evaluat*[tw] OR ‘evaluation tool’ [tw] OR ‘evaluation method’ [tw] OR apprais*[tw] OR ‘appraisal tool’ [tw] OR ‘appraisal method’ [tw] OR intervent* OR risk*[tw] OR tool*[tw] OR measure*[tw] OR evaluat*[tw] OR ‘nursing intervention’[tw] OR ‘psychosocial intervention’[tw] OR ‘sensory intervention*’[tw] OR ‘sensory regulation’[tw] OR ‘Outcome and Process Assessment, Health Care’[Mesh] OR ‘Risk Assessment’[Mesh] OR ‘Healthcare Failure Mode and Effect Analysis’[Mesh] OR ‘Psychosocial Intervention’[Mesh])
	PsycINFO	(‘nonpharmacological intervention’ OR ‘noninvasive intervention’ OR ‘non‐pharmacological intervention’ OR ‘non‐invasive intervention’ OR managem* OR ‘behavioural intervention*’ OR ‘crisis intervention*’ OR ‘risk assessment*’ OR ‘assessment tool’ OR ‘assessment method’ OR manag* OR ‘risk management*’ OR ‘management tool’ OR ‘management method’ OR evaluat* OR ‘evaluation tool’ OR ‘evaluation method’ OR estimat* OR ‘estimation tool’ OR ‘estimation method’ OR evaluat* OR ‘evaluation tool’ OR ‘evaluation method’ OR apprais* OR ‘appraisal tool’ OR ‘appraisal method’ OR intervent* OR tool* OR ‘nursing intervention’ OR ‘psychosocial intervention’ OR ‘sensory intervention*’ OR ‘sensory regulation’ OR DE ‘Measurement’ OR DE ‘Forensic Assessment’ OR DE ‘Needs Assessment’ OR DE ‘Organisational and Occupational Measures’ OR DE ‘Risk Assessment’ OR DE ‘Threat Assessment’ OR DE ‘Treatment Process and Outcome Measures’ OR DE ‘Management Methods’ OR DE ‘Participative Management’ DE ‘Psychosocial Interventions’ OR DE ‘Cognitive Stimulation Therapy’)
	Cochrane Library	non‐pharmacological NEXT intervention* OR non‐invasive NEXT intervention* OR nonpharmacological NEXT intervention* OR noninvasive NEXT intervention* OR managem* OR behavioural NEXT intervention* OR crisis NEXT intervention* OR risk NEXT assessment* OR assessment NEXT tool OR assessment NEXT method* OR manag* OR risk NEXT management* OR management NEXT tool* OR management NEXT method* OR evaluat* OR evaluation NEXT tool* OR evaluation NEXT method OR estimat* OR estimation NEXT tool* OR estimation NEXT method* OR evaluat* OR evaluation NEXT tool* OR evaluation NEXT method* OR apprais* OR appraisal NEXT tool* OR appraisal NEXT method OR intervent* OR tool* OR nursing NEXT intervention* OR psychosocial NEXT intervention OR sensory NEXT intervention* OR sensory NEXT regulation*
	Scopus	(‘nonpharmacological intervention’ OR ‘noninvasive intervention’ OR ‘non‐pharmacological intervention’ OR ‘non‐invasive intervention’ OR managem* OR ‘behavioural intervention*’ OR ‘crisis intervention*’ OR ‘risk assessment*’ OR ‘assessment tool’ OR ‘assessment method’ OR manag* OR ‘risk management*’ OR ‘management tool’ OR ‘management method’ OR evaluat* OR ‘evaluation tool’ OR ‘evaluation method’ OR estimat* OR ‘estimation tool’ OR ‘estimation method’ OR evaluat* OR ‘evaluation tool’ OR ‘evaluation method’ OR apprais* OR ‘appraisal tool’ OR ‘appraisal method’ OR intervent* OR tool* OR ‘nursing intervention’ OR ‘psychosocial intervention’ OR ‘sensory intervention*’ OR ‘sensory regulation*’)
	Web of science	(‘nonpharmacological intervention’ OR ‘noninvasive intervention’ OR ‘non‐pharmacological intervention’ OR ‘non‐invasive intervention’ OR managem* OR ‘behavioural intervention*’ OR ‘crisis intervention*’ OR ‘risk assessment*’ OR ‘assessment tool’ OR ‘assessment method’ OR manag* OR ‘risk management*’ OR ‘management tool’ OR ‘management method’ OR evaluat* OR ‘evaluation tool’ OR ‘evaluation method’ OR psychiat* OR ‘estimation tool’ OR ‘estimation method’ OR evaluat* OR ‘evaluation tool’ OR ‘evaluation method’ OR apprais* OR ‘appraisal tool’ OR ‘appraisal method’ OR intervent* OR tool* OR ‘nursing intervention’ OR ‘psychosocial intervention’ OR ‘sensory intervention*’ OR ‘sensory regulation’)
Institutional setting (Co)	CINAHL	‘mental health’ OR psychiatr* OR inpatient* OR ‘Inpatient Mental Health’ OR ‘Inpatient psychiatr*’ OR ‘Forensic Mental Health’ OR ‘Foster Care’ OR ‘foster home’ OR ‘detention center*’ OR ‘residential care’ OR ‘children* home’
	PubMed	(‘mental health’[tw] OR psychiatr*[tw] OR ‘inpatient mental health’[tw] OR ‘inpatient psychiatr*’[tw] OR ‘forensic mental health’[tw] OR ‘foster care’[tw] OR ‘foster home’[tw] OR ‘detention center*’[tw] OR ‘residential care’[tw] OR ‘children* home’[tw] OR ‘Hospitals, Psychiatric’[Mesh] OR ‘Forensic Psychiatry’[Mesh] OR ‘Foster Home Care’[Mesh] OR ‘Inpatients’[Mesh])
	PsycINFO	(‘mental health’ OR psychiatr* OR inpatient* OR ‘Inpatient Mental Health’ OR ‘Inpatient psychiatr*’ OR ‘Forensic Mental Health’ OR ‘Foster Care’ OR ‘foster home’ OR ‘detention center*’ OR ‘residential care’ OR ‘children* home’ OR DE ‘Institutionalisation’ OR DE ‘Hospitalisation’ OR DE ‘Incarceration’ OR DE ‘Psychiatric Hospitals’ OR DE ‘Psychiatric Units’ OR DE ‘Forensic Psychiatry’ OR DE ‘Foster Care’ OR DE ‘Residential Care Institutions’ OR DE ‘Halfway Houses’ OR DE ‘Orphanages’ OR DE ‘Community Mental Health Centers’ OR DE ‘Psychiatry’ OR DE ‘Addiction Psychiatry’ OR DE ‘Adolescent Psychiatry’ OR DE ‘Community Psychiatry’ OR DE ‘Consultation Liaison Psychiatry’ OR DE ‘Orthopsychiatry’ OR DE ‘Social Psychiatry’ OR DE ‘Transcultural Psychiatry’)
	Cochrane Library	Mental NEXT health OR psychiatr* OR inpatient* OR Inpatient NEXT Mental NEXT Health OR Inpatient NEXT psychiatr* OR Forensic NEXT Mental NEXT Health OR Foster NEXT Care OR foster NEXT home OR detention NEXT center* OR residential NEXT care OR children* NEXT home
	Scopus	(‘mental health’ OR psychiatr* OR inpatient* OR ‘Inpatient Mental Health’ OR ‘Inpatient psychiatr*’ OR ‘Forensic Mental Health’ OR ‘Foster Care’ OR ‘foster home’ OR ‘detention center*’ OR ‘residential care’ OR ‘children* home’)
	Web of science	(‘mental health’ OR psychiatr* OR inpatient* OR ‘Inpatient Mental Health’ OR ‘Inpatient psychiatr*’ OR ‘Forensic Mental Health’ OR ‘Foster Care’ OR ‘foster home’ OR ‘detention center*’ OR ‘residential care’ OR ‘children* home’)

### 3.3 Study Selection

The screening was carried out independently and blinded by two authors (LV, MB) in Covidence. During the screening, a few opposite decisions arose, which were discussed together, and the opinion of the last author (TL) was requested in order to reach a consensus.

### 3.4 Quality Appraisal

The quality of the included studies was appraised using the Mixed Methods Appraisal Tool (MMAT). MMAT enabled the quality assessment of quantitative, qualitative and mixed‐method studies with a single tool. Studies are assessed with seven questions, to which the answers are ‘yes,’ ‘no’ or ‘can't tell’ (Hong et al. 2018). Two authors (LV, MB) made the quality assessment independently. Another researcher (TL) compared the assessments and made the final decision in the case of disagreements between individual judgements.

### 3.5 Data Extraction

Data extraction was made by authors (LV, MB) in Covidence. Consensus judgements were made by the same authors. One article was extracted using a translation program and then reviewed by a native Chinese‐speaking author (YL). The disagreements between individual judgements were discussed, and another author's (TL) opinion was requested for the final decision on extractions.

Detailed information was provided based on the Template for Intervention Description and Replication (TIDieR) (Hoffmann et al., 2014) from the perspective of implementing the new intervention, among others, in light of the results of this review. Instruments and strategies were extracted for violence risk assessment and management according to research questions one and two: (1) short‐term violence risk assessment and (2) risk management strategies in institutional settings. In addition, the extraction aimed at different outcomes (research question three) related to: (1) the effectiveness of violence risk management, (2) the reduction of coercive measures (formal/informal) information, (3) the decrease in risk of violence, (4) any psychometric properties related to the measures, (5) other outcomes (e.g., costs, adverse events, quality of life, injuries) and (6) qualitative feedback (e.g., benefits/disadvantages). General information from the study was obtained, including the authors' names, publication date and country. The focus was on the study's aim, design and participant details, such as the total number of participants, voluntary treatment of adolescents and recruitment method. In addition, outcome measures, intervention names and main results were extracted. Available statistical information about content validity, inter‐rater reliability and predictive validity was extracted. Data about the instrument's feasibility was extracted where available.

## 4 Results

### 4.1 Study Selection Results

**FIGURE 1:**
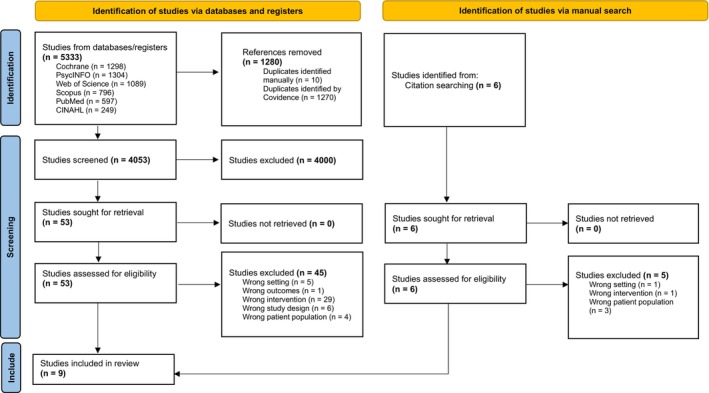
PRISMA flow diagram of the study selection process.

A total of 5339 records were identified from the electronic databases (*n* = 5333) and manual citation searching (*n* = 6). After duplicates (*n* = 1280) were removed, 4053 records remained for the title and abstract level screening. At the title and abstract level, 4000 records were excluded. During the full‐text phase, 53 full‐text studies were assessed for eligibility and 45 studies were excluded. A total of nine studies were included in the review (Chu et al. 2012; Dutch and Patil 2019; Eisenstein et al. 2023; Faay et al. 2013; Kasinathan et al. 2015; Laake et al. 2025; Maguire et al. 2024; Pan et al. 2019; Roaldset et al. 2023). The screening process is presented in the flow chart below (Figure 1).

### 4.2 Study Characteristics

The study characteristics from the included studies are summarised in Table 2. Selected studies were conducted in the following institutions: youth correctional institutions in Singapore (Chu et al. 2012), locked acute care inpatient psychiatric units in the US (Dutch and Patil 2019), adolescent inpatient psychiatric units in the US (Eisenstein et al. 2023), psychiatric units in a university hospital in the Netherlands (Faay et al. 2013), an adolescent forensic psychiatric unit in Australia (Kasinathan et al. 2015; Maguire et al. 2024), the Emergency Unit in the Department of Child and Adolescent Psychiatry, Norway (Roaldset et al. 2023), adolescent inpatient psychiatry and residential youth care child protective services, Norway (Laake et al. 2025) and a mental health department in China (Pan et al. 2019). Of the nine studies included, six used a prospective study design (two were naturalistic observational, one a retrospective study, two were validation studies and one had no further information), two were cross‐sectional studies and one was a two‐phase pilot study. The studies included a total of 1642 adolescents (10–517 per study).

**TABLE 2:** The study characteristics.

				Participants			
Study (author, year, country)	Design	Aim	Outcome measures	Patient description; total sample (*n*), sex ratio (*n*/%), mean age (SD/range), diagnosis, treatment status)	Staff description; total sample (*n*), professional title (*n*/%)	Intervention	Main results
Chu et al. (2012/Singapore)	Prospective quantitative study	To investigate short‐term predictive validity of a dynamic risk assessment measure (DASA) for institutional aggression in a sample of young offenders and to examine differences in predictive accuracy of the DASA between male and female youth offenders	Psychometric properties to predict aggressive episode	*n* = 49 Sex: Male (*n* = 29, 59%), Mean age: 15.42 (SD 1.37, range 12.22–18.24) Dg: N/A Treatment status: involuntary	N/A	DASA	Predictive Validity: Total score significantly predicted any aggressive episode in the next 24 h (odd ratios [OR] = 1.29, 95% confidence interval [95% CI] = 1.07–1.55, *p* < 0.01)
Dutch and Patil (2019/US)	Prospective validation study	To evaluate the interrater reliability and validate a tool to determine if aggressive behaviour on an inpatient psychiatric unit can be better predicted in an ongoing and evolving state with DASA‐YV	Predictive validity, interrater reliability & qualitative feedback	*n* = 127, Sex: N/A, Age: 6–18 years), Dg: all diagnoses were included, Treatment status: N/A	Nursing staff, N/A	DASA‐YV	Inter‐rater reliability: The overall Kappa was 0.79 at 95% CI of 0.70–0.89, and significant as *p* < 0.001. Predictive validity: The AUC for the DASA‐YV as a measure of predictive validity ranged from 0.84 (Physical aggression against other people) to 0.92 (Verbal aggression against other people) Feasibility: (% responses agree) Can do in < 6 min (80%), helps record aggression (100%), useful daily (100%), helps observe better (80%), can use on all patients (60%), predicts < intuition alone (80%), training was adequate (80%), had time to use DASA‐YV (80%), easy to use (80%)
Kasinathan et al. (2015/Australia)	Prospective validation study	To examine the predictive validity of the DASA: Youth Version (DASA:YV) with youth‐specific items, in young offenders hospitalised with a mental illness	Predictive validity and clinical utility	*n* = 35, Sex: male 97%, Age: 14–21 years, Dg: 94% had a mental illness, 83% schizophrenia, 11% bipolar disorder, 89% conduct disorder, Treatment status: Involuntary	Nursing staff, N/A	DASA‐YV	Predictive validity: The AUC was 0.90 (95% CI = 0.86–0.95, *p* < 0.01) A score of 1 or higher maximised sensitivity (65.9%) and specificity (81.1%) in predicting any aggression
Maguire et al. (2024/Australia)	Retrospective study	To re‐analyse an existing data set using contemporary robust data analytic procedures to examine the predictive validity of the DASA‐YV, and to determine risk bands	Predictive validity	*n* = 35 Sex: 34 male, 1 female Age: 14–21 years Dg: 94% had a mental illness, 83% schizophrenia, 11% bipolar disorder, 89% conduct disorder Treatment status: Involuntary	Nursing staff, N/A	DASA‐YV	Predictive validity: DASA three AUC = 0.8521, DASA four AUC = 0.8525 DASA 1–3 Odd ratios [OR] = 1.983 (95% confidence interval [CI]: 1.112, 3.537; *p* < 0.01) and DASA 4–11 OR = 4543 (95% CI; 2.834, 7.284)
Eisenstein et al. (2023/US)	Two‐phase pilot study	To develop and evaluate the Pedi‐BEWS as a screening tool to prompt quick appropriate interventions and to decrease incidents of restraint and seclusion and/or administration of psychotropic medications	Reliability, content‐ & construct validity	*n* = 456, Sex: N/A, Age: < 18 years, Dg: all, Treatment status: N/A	*n* = 21, Nursing staff	Pedi‐BEWS	Content validity: The overall CVI for three‐item was 0.73. Simplicity: 0.98. clarity: 0.97. Necessity and relevance: 0.98. Construct validity ROC curve: 0.890 cognition, 0.959 affect, 0.951 behaviour Internal consistency: The Cronbach's alpha was 0.99. Inter‐rater reliability (ICC) was 0,78, 077, 078 and overall 0.83
Faay et al. (2013/the Netherlands)	Cross sectional study	To determine the interrater reliability and clinical utility of the Kennedy Axis V by MHN in clinical practice at three psychiatric units in the Netherlands	Interrater reliability & clinical utility	*n* = 10, Sex: male 50%, Age: mean 16.3 SD 2.2 years, Dg: Psychotic disorder, mood disorder, ASD, substance abuse, multiple dg. & other disorders Treatment status: Involuntary 40%	*n* = 12 (*n* = 23), Master's degree *n* = 3, RN *n* = 16, LPN *n* = 4	Kennedy Axis V	The overall inter‐rater reliability was 0.79 Feasibility: Supports more objective assessment, time‐consuming in the beginning. Needs frequent training to refine age‐specific ratings and maintain routine
Laake et al. (2025/Norway)	Naturalistic, prospective observational study	To assess the predictive accuracy of V‐RISK‐Y for youth in two types of acute institutions providing 24‐h care for youth, namely acute inpatient psychiatric units and acute residential youth care units under child protective services	Internal consistency & predictive validity	*n* = 517 Age: mean 15.0 (girls) and 15.6 (boys) Dg: suicidal (44.6%), eating disorders (6.3%), behaviour (17.0%), assessment (9.7%), care/custody (16%), other (6.5%) Treatment status: Voluntary/involuntary	Physicians/psychiatrists, psychologists and milieu therapists (e.g., nurses, social workers). N/A	V‐RISK‐Y	The internal consistency: Cronbach's alpha was 0.791 (95% confidence interval [CI]: 0.76, 0.82; *p* < 0.001) Predictive validity: The Area under the curve [AUC] was 0.762 (95% CI: 0.700, 0.824, *p* < 0.001) Other predictive values were sensitivity 0.83, and specificity 0.52. Positive predictive value [PPV] was 0.17 and negative predictive value [NPV] of 0.96.
Roaldset et al. (2023/Norway)	Naturalistic prospective observational study	To test the predictive validity and feasibility of a pilot version of the V‐RISK‐Y in an inpatient emergency psychiatric unit	Internal consistency, inter‐rater reliability & predictive validity	*n* = 67, Sex: male 18%, Age: mean age 15.5 years for female, 16.1 years for male, Dg: Psychoses, eating disorders, autism spectrum disorders, affective disorders and anxiety disorders, Treatment status: N/A	*n* = 70, Ward staff members	V‐RISK‐Y	The internal consistency (Cronbach's alpha) for the summed score of all 12 items was 0.78 (95% CI 0.69–0.85, *p* < 0.001). Inter‐rater reliability: The ICC for the summed total score was 0.51 Predictive value: The AUC for the V‐RISK‐Y was recalculated when Do not know = 0, showing a decrease from 0.762 (95% CI = 0.57–0.96), *p* = 0.006 to 0.741 (95% CI = 0.54–0.94), *p* = 0.012. Other predictive values were sensitivity 0.82, specificity 0.75, PPV 0.39 and NPV 0.95
Pan et al. (2019/China)	Cross‐sectional study	To test the validity and reliability of the Chinese version of the BVC in Chinese children and adolescents with mental disorder	Validity & reliability	*n* = 346, Sex: male 55.6%, Age: 4–11 years (12.4%), 12–16 years (87.6%), Dg: Schizophrenia and bipolar 1 disorder, Treatment status: N/A	*n* = 6, Experts in the field of psychiatry and mental health	BVC	The content validity: 0.91. There was a moderate positive correlation between the total score and the score of the Achenbach Child Behaviour Checklist‐child behaviour Predictive validity: Chinese version of the BVC scale was effective in children. It has good predictive validity among adolescents with mental illness

Abbreviations: BVC, Broset‐Violence‐Checklist; DASA, Dynamic Appraisal of Situational Aggression; DASA‐YV, Dynamic Appraisal of Situational Aggression—Youth Version; Dg, diagnosis; N/A, not available; Pedi‐BEWS, Paediatric Behavioural Early Warning Scale; V‐RISK‐Y, Violence Risk Screening for Youth.

### 4.3 Quality of Included Studies

The results of assessing the quality of the included studies are presented in Table 3.

**TABLE 3:** Quality appraisal of the included studies using the MMAT.

References	Screening questions		Qualitive studies					Quantitative descriptive studies					Mixed‐methods studies					Total points
Chu et al. (2012)	Y	Y						Y	CT	Y	Y	Y						6/7
Dutch and Patil (2019)	Y	Y						Y	Y	Y	Y	Y						7/7
Eisenstein et al. (2023)	Y	Y						Y	Y	Y	CT	Y						6/7
Faay et al. (2013)	Y	Y	Y	Y	Y	Y	Y	Y	CT	Y	Y	Y	Y	Y	Y	Y	Y	16/17
Kasinathan et al. (2015)	Y	Y						Y	CT	Y	Y	Y						6/7
Maguire et al. (2024)	Y	Y						Y	CT	Y	Y	Y						6/7
Laake et al. (2025)	Y	Y						Y	Y	Y	Y	Y						7/7
Pan et al. (2019)	Y	Y						Y	Y	Y	Y	Y						7/7
Roaldset et al. (2023)	Y	Y	Y	Y	Y	Y	CT	Y	Y	Y	Y	Y	Y	Y	CT	CT	N	13/17

Abbreviations: CT, Can't tell based on the article; N, No; Y, Yes.

Seven of the studies were quantitative, and two mixed‐method studies also had quantitative descriptive components. All the studies received ratings ranging from 4 to 5 out of 5. However, there may be limitations in generalising the findings to broader populations due to the relatively small sample sizes (Chu et al. 2012). Additionally, there was an underrepresentation of females, with only one female participant compared to 34 males, indicating that the sample may not fully represent the broader adolescent population (Kasinathan et al. 2015; Maguire et al. 2024). It is worth noting that the studies involved regular assessments of adolescents during their hospitalisation, which ensures that the entire sample is accounted for. However, in the study by Eisenstein et al. (2023), some assessments were conducted weekly while others were done daily.

The included mixed‐method studies were rated 13/15 and 11/15. The studies' assessments were rated 4/5 and 5/5 for both their qualitative and quantitative components. (Faay et al. 2013; Roaldset et al. 2023). The interpretation of results seemed to be well supported by both quantitative and qualitative data, although limitations in sampling and adherence could have been addressed more clearly (Faay et al. 2013). The interpretation of the qualitative data mainly provided context rather than being deeply analysed concerning the quantitative findings. The study does not explicitly address any divergences between quantitative and qualitative results. There is a brief mention of qualitative feedback from staff regarding the feasibility of the V‐RISK‐Y. However, the coherence seemed limited as the qualitative data did not appear to be extensively integrated into the overall findings or analysis (Roaldset et al. 2023).

### 4.4 Short‐Term Violence Risk Assessment Instruments

First, we summarised what instruments were identified and used among adolescents in the studies, and then described the details of the assessments of each instrument. Second, we grouped and presented each risk assessment instrument. DASA‐YV was featured in three studies, and V‐RISK‐Y was featured in two studies.

#### 4.4.1 TIDieR Summary

Six assessment instruments used among adolescents for short‐term violence risk were identified from the selected studies: The Dynamic Appraisal of Situational Aggression (DASA; Chu et al. 2012), the Youth Version of DASA (DASA‐YV; Dutch and Patil 2019; Kasinathan et al. 2015; Maguire et al. 2024), the Violence Risk Assessment Checklist for Youth (V‐RISK‐Y; Laake et al. 2025; Roaldset et al. 2023), the Kennedy Axis V (Faay et al. 2013), the Paediatric Behavioural Early Warning Scale (Pedi‐BEWS; Eisenstein et al. 2023) and the Brøset Violence Checklist (BVC; Pan et al. 2019). The instruments used for violence risk assessments were either actuarial (DASA, DASA‐YV), Structured Professional Judgement (SPJ) (The Kennedy Axis V) or included both actuarial and SPJ approaches (BVC, Pedi‐BEWS, V‐RISK‐Y). All instruments measured dynamic risk variables. All assessment instruments are described using the TIDieR checklist and are elaborated in Table 4.

**TABLE 4:** Data extraction using Template for Intervention Description and Replication (TIDieR) (Hoffmann et al. 2014).

Brief name	DASA	DASA‐YV			Pedi‐BEWS	Kennedy Axis V	BVC	V‐RISK‐Y	
Author	Chu et al. (2012)	Dutch and Patil (2019)	Kasinathan et al. (2015)	Maguire et al. (2024)	Eisenstein et al. (2023)	Faay et al. (2013)	Pan et al. (2019)	Laake et al. (2025)	Roaldset et al. (2023)
Why	Current evidence does not support capacity of medium to long‐term risk assessment to measure change in risk state on daily basis	Four additional items were added from different methods and studies outcomes to original validated DASA to create the youth version	Current evidence for adolescents in institutional setting are designed to predict violence over the medium to long‐term and such measures lack the capacity to measure change on daily basis. Added factors may weaken accuracy of adult derived measures	Other violence risk assessment tools for adolescents in institutional setting are designed to predict violence over the medium to long‐term and such measures lack the capacity to measure change on a daily basis	Current screening methods available do not assess the mental status of adolescent inpatients as well as violent behaviours as means for detecting early signs or symptoms of potential distress	Kennedy Axis V was developed to refine the Global Assessment of Functioning, which is incorporated in Axis V of DSM‐IV	According to previous evidence, violent incidence is common in child & adolescent psychiatric wards and therefore there is need to implement effective risk assessment method	Developed for use in acute youth institutions and designed to be quick and easy to use, even for untrained staff, in situations where conducting comprehensive violence risk assessments are not feasible	No other screener or short‐term instruments for assessing the risk of violence in youths have been validated for acute use. Given this lack, some emergency psychiatric departments for adolescents have used the V‐RISK‐10
What (material)	No further details about material or form 7 items: Negative attitudes, impulsivity, irritability, verbal threats, sensitive to perceived provocation, easily angered when requests are denied and unwillingness to follow directions Each item scored 0 or 1 for presence of the specific behaviour over the past 24 h, where the individual's usual behaviour while being non‐violent is scored as 0. Total possible score 0–7 Final risk (The SPJ rating): Low, Medium or High	No further details about material or form 11 items: Irritability, impulsivity, unwillingness to follow directions, sensitivity to perceived provocation, easily angered when requests are denied, negative attitudes, verbal threats, anxious or fearful, low empathy/remorse, significant peer rejection and outside stressor Each item scored 0 for no change and 1 for demonstration of the specific behaviour over the past 24 h, for a total possible score of 0 to 11 Final risk (The SPJ rating): Low, Medium or High			Described as form what can be hold in hands. No further details 3 items: Cognition, affect, behaviour. Each construct has a maximal value of 3 and minimum value 0. Score indicates state of risk (3 = high risk, 0 = no risk). Score was based on nurses observation. Each patient's behavioural/mental changes of unmet needs or potential distress were rated	No further details about material or form Manual provides brief descriptions of the severity scores to support the completion of the subscales. 7 items: Psychological impairment (mood changes, psychotic symptoms); social skills; violence; activities of daily living and occupational skills; substance misuse; medical impairment; and ancillary impairment	No further details about material or form. Translated into Chinese. 6 items: Confusion, irritability, roughness, physical threats, verbal threats, and attacking objects 0 points divided as low risk, 1 to 2 points as medium risk, and 3 to 6 points as high risk	Form is accessible from internet, manually filled in paper 12 items: Violent acts; violent threats; substance abuse; severe mental symptoms; disruptive or impulsive behaviour or behavioural disorders; lack of insight; suspicion; lack of empathy; unrealistic plans; stress vulnerability, childhood adversities; and adolescent's/parent's own assessment All 12 scored: (1) No = do not fit, (2) Maybe or is present in moderately, (3) Yes = is present, and (4) Do not know = too little or conflicting information Final risk (The SPJ rating): Low, Moderate, or High risk	Reported as a form, but there's no further details about concrete material 12 items: Violent acts; violent threats; substance abuse; severe mental symptoms; disruptive or impulsive behaviour or behavioural disorders; lack of insight; suspicion; lack of empathy; unrealistic plans; stress vulnerability, childhood adversities; and adolescent's/parent's own assessment All 12 scored: (1) No = do not fit, (2) Maybe or is present in moderately, (3) Yes = is present and (4) Do not know = too little or conflicting information Final risk (The SPJ rating): Low, Moderate or High risk
What (procedures)	Staff received training. The criteria for scoring were discussed in detail	Coaching was provided. Training were provided with patients and peer comparison	Staff were trained Staff were not instructed to act in any particular way if there were elevated scores	Staff rate each youth daily and final risk rating were matched with the subsequent day's recording of aggression to examine predictive validity	Can be used with minimal training, but there is no further details about training or supportive material	No further details of training	Training were provided, no further details of content of training	Prior the implementation, staff participated in a 3‐h introduction seminar. Scoring based on available information (e.g., medical records)	No supervision or follow‐up; the V‐RISK‐Y is self‐instructing and with all the necessary information on the form. Scored by the staff after clinical admission interview
Who provided	Supervision staff. No further details about characteristic of staff	Trained nurses			Registered nurses	Trained nurses	Trained nurses	Healthcare or social care staff	Ward staff. No further details about characteristic of staff
How	Individually. No further details	Individually on each patient every 24 h during the patient stay	Individually based on observation	Individually on each youth based on observation	Individually based on observation	Individually. Ratings addressed on reports and noted in the patient's file	Individual observation every 8 h in every shift	Scoring interdisciplinary if feasible. The youth and/or caregivers were not present during scorings	Individually without patients and/or patients' parents No further details
Where	High‐security correctional institution. No further details	Child and adolescent psychiatric inpatient unit. No further details	Adolescent forensic psychiatric inpatient unit. No further details	Adolescent forensic mental health unit providing acute care and rehabilitation to adolescents	Adolescent psychiatric inpatient unit. No further details	Psychiatric inpatient unit. No further details	Child and adolescents psychiatric inpatient ward. No further details	Emergency psychiatric inpatient units for youth and residential youth care units	Psychiatric emergency unit. No further details
When and how much	Daily in the 24 h prior to assessment. No further details	Every 24 h during the patient stay between the hours of 1 p.m. and 3 p.m.	Daily, 9 p.m. for each inpatient, scoring each item for its presence or absence in the 24 h prior	Daily. No further details	Twice in 24 h: during day and evening shifts and can be utilised for entire length of stay in hospital. No further details	4 of the subscales were scored daily, all eight subscales were scored weekly for each patient	Three times per day. After every shift	Typically scored following the intake session, where staff had an opportunity to collect relevant data (e.g., assess youth perception of violence risk)	After clinical admission interview. One time. No further details
Tailoring	N/A	N/A	N/A	N/A	N/A	N/A	N/A	N/A	N/A
Modification	N/A	N/A	N/A	N/A	N/A	N/A	N/A	N/A	N/A
How well (planned)	The staff members were blind to the participants' behaviours that occurred in subsequent shifts	24 were included in inter‐rater reliability phase and 103 in the validation study	Assessment was planned to conduct daily from 03/11 to 11/13 for each patient	N/A	N/A	N/A	N/A	One admission per youth was included in analyses, so that any person would only count once in analyses	Assessment was planned to conduct to all patient and assess all of the 12 items
How well (actual)	Low staff‐to‐young person ratio (~1:30) may have meant staff were not able to ensure valid and accurate assessment	47 daily risk assessments collected from 24 patients. 465 daily risk assessment from 83 patients were completed	A total of 4440 DASA‐YV ratings were completed	N/A	N/A	N/A	N/A	A total of 67 patients (73%) were assessed. Sixteen forms were fully completed according to the instructions	67 patients (73%) were assessed. 16 forms were completed whole in accordance with the instructions

Abbreviations: BVC, Broset‐Violence‐Checklist; DASA, Dynamic Appraisal of Situational Aggression; DASA‐YV, Dynamic Appraisal of Situational Aggression—Youth Version; DSM‐IV, the fourth edition of Diagnostic and Statistical Manual of Mental Disorders; N/A, Not Available; Pedi‐BEWS, Paediatric Behavioural Early Warning Scale; SPJ, Structured Professional Judgement; V‐RISK‐Y, Violence Risk Screening for Youth.

The assessment was conducted by nursing staff, for example, practical and/or registered nurses (Dutch and Patil 2019; Eisenstein et al. 2023; Faay et al. 2013; Kasinathan et al. 2015; Laake et al. 2025; Maguire et al. 2024; Pan et al. 2019), and both supervision staff (Chu et al. 2012) and ward staff (Roaldset et al. 2023) with no further details about degrees/qualifications. Scoring was based on observations by the professionals in all of the studies. Assessments were made at different intervals, for example, three times per day (Pan et al. 2019), twice per day (Eisenstein et al. 2023), daily during data collection (Roaldset et al. 2023), daily (Chu et al. 2012; Dutch and Patil 2019; Kasinathan et al. 2015; Laake et al. 2025; Maguire et al. 2024); in addition, some parts of the instrument were collected daily and some parts weekly (Eisenstein et al. 2023).

#### 4.4.2 DASA

DASA is a risk assessment instrument that evaluates the likelihood of short‐term aggression within the next 24 h in mental health units. The total score (i.e., actuarial rating) predicts the risk of violence within 24 h. The instrument includes seven items: negative attitudes, impulsivity, irritability, verbal threats, sensitivity to perceived provocation, being easily angered when requests are denied and unwillingness to follow directions. Current evidence suggests that medium‐ to long‐term risk assessment measures are inadequate for capturing daily risk fluctuations, a crucial aspect of management and treatment decisions in institutional settings. (Ogloff and Daffern 2006). Before the study, supervisory staff members received training on the principles of violence risk assessment and the scoring of DASA. The criteria for scoring were discussed in detail during the training session to ensure clarity. (Chu et al. 2012).

#### 4.4.3 DASA‐YV

DASA‐YV was used in three studies and is a predictive structured measurement instrument developed specifically for the youth population (Dutch and Patil 2019; Kasinathan et al. 2015). The instrument consists of 11 items including the seven original DASA items and four youth‐specific items added to DASA‐YV: anxiety or fear, low empathy/remorse, significant peer rejection and outside stressors (Dutch and Patil 2019). Each item is scored (i.e., actuarial rating) 0 for no change and 1 for the demonstration of specific behaviour over the past 24 h, for a total possible score ranging from 0 to 11 (Dutch and Patil 2019; Kasinathan et al. 2015; Maguire et al. 2024).

#### 4.4.4 The V‐RISK‐Y

The V‐RISK‐Y was used in two of the included studies. The Youth version of V‐RISK‐10 includes 12 risk factors measuring dynamic variables: previous or current violent acts, previous or current violent threats, previous or current substance abuse, previous or current severe mental symptoms, disruptive impulsive behaviour or behavioural disorders, lack of insight, suspicion, lack of empathy, unrealistic plans, stress vulnerability, childhood adversities (previous or current) and the adolescent's or parent's own risk assessment. Each item is scored (i.e., actuarial rating) based on its presence and relevance of the item for future violence: (1) No = does not fit, (2) Maybe = present to a moderate degree, (3) Yes = is present and (4) Do not know = insufficient or conflicting information. The SPJ rating involves the final risk assessment categorised as Low, Moderate or High risk. (Laake et al. 2025; Roaldset et al. 2023).

#### 4.4.5 The Kennedy Axis V

The Kennedy Axis V is a measurement instrument used in studies to assess short‐term violence risk (Kennedy 2003). This routine outcome measurement was developed to enhance the Global Assessment of Functioning (GAF) part of Axis V in the DSM‐IV, consisting of seven subscales measuring dynamic variables: psychological impairment (including mood changes and psychotic symptoms), social skills, violence, activities of daily living, occupational skills, substance misuse, medical impairment and ancillary impairment. In current knowledge, the instrument is based on actuarial rating (Faay et al. 2013).

#### 4.4.6 Pedi‐BEWS

Pedi‐BEWS is a screening instrument that employs a four‐point scoring system to measure warning signs and reduce restraints (Eisenstein et al. 2023). Pedi‐BEWS evaluates three areas: cognition, affect and behaviour. Each area has a maximum value of 3 and a minimum of 0. The total score (i.e., actuarial rating and SPJ) indicates the risk level (3 = high risk, 0 = no risk) (Eisenstein et al. 2023).

#### 4.4.7 BVC

BVC evaluates six items: confusion, irritability, boisterousness, physical threats, verbal threats and attacks on objects occurring within 24 h before a violent incident. The total score (i.e., actuarial and SPJ rating) reflects the risk level and likelihood of future violent behaviour: 0 points indicate low risk, 1–2 points indicate medium risk and 3–6 points indicate high risk (Almvik et al. 2000). BVC's Chinese version has been tested in this study underscoring the need for effective risk assessment instruments (Pan et al. 2019).

### 4.5 Short‐Term Violence Risk Management Strategies and Outcomes

This review found no studies addressing short‐term violence risk management strategies or outcomes of these methods.

### 4.6 Risk Assessment Outcomes

First, we summarised the predictive validity. The Area Under the Curve (AUC) is a commonly used predictive validity assessment, as it is not dependent on the base rate of violence. The scores in AUC vary from 0 (no prediction) to 0.5 (chance prediction) to 1.0 (excellent prediction). (Douglas et al. 1999). Second, we summarised content validity, consistency and inter‐rater reliability if available. Third, feasibility was presented if available. No other outcomes were identified from the studies. The psychometric properties of the instruments are presented in Table 5.

**TABLE 5:** Psychometric properties of the identified methods.

DASA	Chu et al. (2012)	Predictive validity: any aggression in the next 24 h (OR = 1.29, 95% CI = 1.07–1.55, *p* < 0.01) and 48 h (OR = 1.34, 95% CI = 1.16–1.54, *p* < 0.001); and with males, significantly prediction of any aggression in the following 24 h (OR = 1.24, 95% CI = 1.02–1.51, *p* < 0.05) and 48 h (OR = 1.29, 95% CI = 1.11–1.49, *p* < 0.01) and with female subsample no significant prediction in the next 24 and 48 h
DASA‐YV	Dutch and Patil (2019)	Predictive validity (AUC): from 0.84 (Physical aggression against other people) to 0.92 (Verbal aggression against other people) Inter‐rater reliability: The overall Kappa was 0.79 at 95% CI of 0.70 to 0.89, and significant as *p* < 0.001
DASA / DASA‐YV	Kasinathan et al. (2015)	Predictive validity (AUC): DASA‐YV (AUC = 0.754), and DASA (*χ* ^2^ = 6.04; *p* = 0.014). Other predictive values on DASA‐YV: sensitivity (65.9%) and specificity (81.1%). Both the DASA‐YV and DASA had greater predictive validity than the ‘final risk rating’, a clinical judgement of risk (respectively, the *χ* ^2^ = 14.57 and *p* < 0.001, and *χ* ^2^ = 8.28 and *p* = 0.004)
DASA‐YV	Maguire et al. (2024)	Predictive validity (OR): DASA 1–3, OR = 1.983 (95% CI; 1.112, 3.537), for DASA scores of 4–11, OR 4.543 (95% CI; 2.834, 7.284)
BVC	Pan et al. (2019)	Predictive validity (AUC): 0.93. When the cut‐off was 2, the sensitivity was 83.7% and the specificity was 89.0% Content validity: 0.91. There was a moderate positive correlation between the total score and the score of the Achenbach Child Behaviour Checklist‐child behaviour Internal consistency (The Cronbach's α coefficient): 0.90 and the item total correlations were 0.60–0.81 (*p* < 0.01)
Kennedy Axis V	Faay et al. (2013)	Inter‐rater reliability (ICC): 0.79
Pedi‐ BEWS	Eisenstein et al. (2023)	Content validity (CVI) for three‐item was 0.73. Simplicity: 0.98, clarity: 0.97. Necessity and relevance: 0.98 Construct validity (ROC): 0.890 cognition, 0.959 affect, 0.951 behaviour Internal consistency (The Cronbach's alpha): 0.99 Inter‐rater reliability (ICC): 0.78, 0.77, 0.78 and overall 0.83
V‐RISK‐Y	Laake et al. (2025)	Predictive validity (AUC): 0.762 (95% CI: 0.700, 0.824, *p* < 0,001). Maintaining sensitivity of > 80%, the best cutoff for V‐RISK‐Y for the full sample was 12.5. Other predictive values were: sensitivity 0.83, and specificity of 0.52, PPV of 0.17 and NPV of 0.96. Logistic regression analyses (OR = 3.281; CI: 1.89, 5.70; *p* < 0.001) The internal consistency: 0.791 (95% CI: 0.76, 0.82; *p* < 0.001)
V‐RISK‐Y	Roaldset et al. (2023)	Predictive validity (AUC): Recalculated when Do not know = 0, showing a decrease from 0.762 (95% CI = 0.57–0.96), *p* = 0.006 to 0.741 (95% CI = 0.54–0.94), *p* = 0.012. Other predictive values were sensitivity 0.82, specificity 0.75, PPV 0.39 and NPV 0.9 Internal consistency (Cronbach's alpha): 0.78 (95% CI 0.69–0.85, *p* < 0.001) Inter‐rater reliability (ICC): 0.51

Abbreviations: AUC, Area Under the Curve; BVC, Broset‐Violence‐Checklist; CI, Confidence Interval; DASA, Dynamic Appraisal of Situational Aggression; DASA‐YV, Dynamic Appraisal of Situational Aggression—Youth Version; ICC, Intraclass Correlation Coefficient; NPV, Negative Predictive Value; OR, Odd Ratios; Pedi‐BEWS, Paediatric Behavioural Early Warning Scale; PPV, Positive Predictive Value; V‐RISK‐Y, Violence Risk Screening for Youth.

#### 4.6.1 DASA

DASA significantly predicted any aggressive episode in the forthcoming 24 h (odd ratios [OR] = 1.29, 95% confidence interval [95% CI] = 1.07–1.55, *p* < 0.01). Odds ratios suggest that for every one‐point increase in the DASA total score, there was a 1.29‐times increased likelihood that adolescents would behave aggressively in the following 24 h (Chu et al. 2012).

#### 4.6.2 DASA‐YV

The predictive validity (AUC) ranged from 0.84 (Physical aggression against other people) to 0.92 (Verbal aggression against other people) (Dutch and Patil 2019) and any form of aggression 0.754 (Kasinathan et al. 2015). When comparing the AUC for any form of aggression, YV had a greater predictive validity than the DASA (*χ* ^2^ = 6.04; *p* = 0.014). The overall Kappa was 0.79 at a 95% CI of 0.70–0.89 and significant at *p* < 0.001 (Dutch and Patil 2019). The odd ratios [OR] of predictive validity varied from 1.983 to 4.543 and the confidence interval [95% CI] from 1.112, 3.537 to 2.834, 7.284 (Maguire et al. 2024). A predictive validity score of 1 or higher on the DASA‐YV maximised sensitivity (65.9%) and specificity (81.1%) in predicting any form of aggression (Kasinathan et al. 2015). According to the feasibility outcomes, respondents reported that DASA‐YV was a useful instrument for recording daily aggression. Most respondents (80%) reported that the instrument was quick to complete, supported their observations, aligned with or enhanced their clinical intuition, came with sufficient training, allowed adequate time for use and was easy to use. Additionally, 60% of respondents believed the instrument could be applied to all youth patients (Dutch and Patil 2019).

#### 4.6.3 The V‐RISK‐Y

The predictive validity (AUC) for the V‐RISK‐Y was recalculated when Do not know = 0, showing a decrease from 0.762 (95% CI = 0.57–0.96, *p* = 0.006) to 0.741 (95% CI = 0.54–0.94, *p* = 0.012) (Roaldset et al. 2023). In re‐analysis, the AUC was 0.762 (95% CI = 0.700–0.824, *p* < 0.001), while maintaining a sensitivity of over 80%. The odds ratios of predictive validity were 3.281 and the confidence interval [95% CI] varied from 1.89 to 5.70; *p* < 0.001. The internal consistency was 0.791 (95% CI = 0.76, 0.82; *p* < 0.001) (Laake et al. 2025). The inter‐rater reliability (ICC) for the total score was moderate (0.51), as the Low‐Moderate/Maybe‐High risk estimate was 0.42 (Roaldset et al. 2023).

The feasibility was mainly rated as good, moderate (*n* = 1), good (*n* = 11) and very good (*n* = 4). Six participants assessed the V‐RISK‐Y to be as useful as the V‐RISK‐10, while ten rated the V‐RISK‐Y as being more useful. Staff found rating the relevance of violence, especially for unknown patients, challenging and time‐consuming. They also mentioned that the concept of relevance required more in‐depth consideration and was difficult to assess in the context of the admission situation. However, all the participants recommended the use of V‐RISK‐Y in emergency adolescent psychiatry. (Roaldset et al. 2023).

#### 4.6.4 The Kennedy Axis V

The overall inter‐rater reliability (ICC) of the Kennedy Axis V was found to be high at 0.79. As regards the feasibility outcomes, the assessment was considered more objective and useful for standard observations and risk assessment. The current risks, strengths and skills of patients should be frequently reassessed. However, participants also noted that the instrument is time‐consuming and requires more frequent training. Some difficulties were reported in scoring, as it felt challenging to relate the content of the manual to the adolescent population according to some nurses. The study revealed that around 70% of nurses found the instrument useful for care planning, nursing reports and multidisciplinary meetings. (Faay et al. 2013).

#### 4.6.5 Pedi‐BEWS

The Content Validity Index (CVI) for all three items, cognition, affect or emotion and behaviour, was found to be 0.73. Initially, the scores for simplicity (0.98) and clarity (0.97) were high. Necessity and relevance received a score of 0.98. Inter‐rater reliability with ICC was 0.78 (cognition), 0.77 (affect) and 0.78 (behaviour). (Eisenstein et al. 2023).

#### 4.6.6 BVC

The predictive validity (AUC) was 0.93. When the cut‐off was 2, the sensitivity was 83.7% and the specificity was 89.0%. The instrument demonstrated a content validity (CVI) of 0.91, and each item level on the scale is as follows: confusion (0.67), irritability (1.00), noise (0.83), verbal threats (1.00), physical threats (1.00) and destructive behaviour (1.00). The inter‐rater reliability (ICC) was 0.87 (*p* < 0.01) for confusion (0.86), irritability (0.85), noise (0.87), verbal threats (0.93), physical threats (0.83) and destructive behaviour (0.87). The internal consistency reliability results showed that the Cronbach α coefficient was 0.897, and the Pearson correlation analysis indicated high item‐total correlations of 0.60–0.81 (*p* < 0.01). (Pan et al. 2019).

## 5 Discussion

This review aimed to identify different short‐term (< 24 h) violence risk assessment instruments and concrete management strategies for adolescents in institutional settings and synthesise the outcomes available from the studies. In this review, nine studies were found that evaluated six different instruments (DASA, DASA‐YV, BVC, Pedi‐BEWS, The Kennedy Axis V, V‐RISK‐Y) regarding short‐term violence risk assessment among adolescents in institutional settings. Notably, only two of these instruments, DASA‐YV (Dutch and Patil 2019; Kasinathan et al. 2015; Maguire et al. 2024) and V‐RISK‐Y (Laake et al. 2025; Roaldset et al. 2023), were specifically designed for adolescent populations. For instance, the DASA was developed in the adult forensic psychiatric ward (Ogloff and Daffern 2006). However, only two instruments (DASA‐YV, V‐RISK‐Y) were explored in more than one research article (Dutch and Patil 2019; Kasinathan et al. 2015; Laake et al. 2025; Maguire et al. 2024; Roaldset et al. 2023), so definite conclusions about which instruments would be suitable for clinical practice cannot be determined based on this review.

This review found no studies addressing short‐term violence risk management strategies or outcomes for these instruments. According to our findings, there is a clear gap in research regarding short‐term violence risk management. Although short‐term risk assessment instruments are considered an important starting point for risk management (Vincent et al. 2012), this may be the reason that violence risk management is not separated into evaluative components in addition to risk assessment. This tradition can also be seen in research with adults. For example, a randomised controlled trial with BVC (Abderhalden et al. 2006) included only a risk assessment instrument as a study intervention, but risk management strategies in general were not specified. However, focusing on early risk management through concrete strategies would be important to avoid recourse to coercive measures. Recently, the focus on adults has shifted to understanding the need for guidance in selecting evidence‐based and non‐coercive management strategies. For instance, an electronic version of the DASA (eDASA+APP) was created to provide intervention suggestions based on the assessment level indicated by the DASA (Maguire et al. 2019).

For this review, we sought information on whether risk‐assessment instruments could reduce coercive measures, decrease violence risk, assess any psychometric properties related to the measures, as well as other outcomes (e.g., quality of life) and qualitative feedback (e.g., benefits). However, we found only outcomes related to psychometric properties, feasibility and qualitative feedback of using the instruments. In total, we discovered five different psychometric properties that were explored in the included studies: inter‐rater reliability (Dutch and Patil 2019; Eisenstein et al. 2023; Faay et al. 2013; Roaldset et al. 2023), content validity (Eisenstein et al. 2023; Pan et al. 2019), construct validity (Eisenstein et al. 2023), internal consistency (Eisenstein et al. 2023; Laake et al. 2025; Roaldset et al. 2023; Pan et al. 2019) and predictive validity (Chu et al. 2012; Dutch and Patil 2019; Kasinathan et al. 2015; Laake et al. 2025; Maguire et al. 2024; Pan et al. 2019; Roaldset et al. 2023). The studies that reported predictive outcomes had AUC scores between 0.5 and 1.0. When comparing the DASA's scoring from 0.50 to 0.69 (Chu et al. 2012) and the youth version of DASA, the DASA‐YV ranged from 0.72 to 0.95 (Dutch and Patil 2019; Kasinathan et al. 2015; Maguire et al. 2024) and the V‐RISK‐Y from 0.762 to 0.762 (Laake et al. 2025; Roaldset et al. 2023); therefore, it is clear that the youth‐specific instruments produced higher predictive values. The lower predictive value ratings in DASA can be a result of different study environments (youth correctional institute) in which the instruments had been originally developed, that is, secure forensic care (Ogloff and Daffern 2006). Therefore, it is important to evaluate which instrument is most suitable in a specific environment. Despite existing research on short‐term instruments in institutional settings, such as inpatient care units (Dutch and Patil 2019; Eisenstein et al. 2023; Faay et al. 2013; Pan et al. 2019), psychiatric emergency units (Laake et al. 2025; Roaldset et al. 2023), forensic psychiatric units (Kasinathan et al. 2015; Maguire et al. 2024) and youth correctional institutes (Chu et al. 2012), there remains a lack of studies that include foster care centres in short‐term risk assessment and management. This needs to be addressed as violence is common among youths in foster care centres (Bergstrom and Hojman 2016).

In selected studies, feasibility outcomes were examined with three instruments (DASA‐YV, Kennedy Axis V and V‐RISK‐Y). Outcomes from these youth‐specific instruments (DASA‐YV and V‐RISK‐Y) received better feedback compared to Kennedy Axis V. With Kennedy Axis V, feasibility feedback provided by nursing staff offers insight not only into the practicality of instruments but also into how these tools align with nursing workflows and therapeutic approaches. Responders felt some items scored did not match developmental and childhood issues in youths; they needed more training on using the scale, and the manual did not provide enough information for rating. However, some nurses found the instrument efficient to use and a good means of observing violent behaviour. (Faay et al. 2013). Most of the participants (63%) rated the V‐RISK‐Y as more useful than the non‐youth version (V‐RISK‐10). In addition, V‐RISK‐Y was rated as time‐consuming and difficult with unknown patients. However, all respondents recommended the use of V‐RISK‐Y. (Roaldset et al. 2023). DASA‐YV was rated very feasible as most of the percentages of the responders' agreement on the statements (e.g., useful on a daily basis) were 80%–100% (Dutch and Patil 2019). Notably, with in‐depth interviews (Dutch and Patil 2019) and a short evaluation form (Roaldset et al. 2023), there were presumably more possibilities to add verbal feedback. However, in the Dutch and Patil (2019) study, feasibility was evaluated using a structured Likert‐scale questionnaire, which may have limited the opportunity for more detailed qualitative feedback, unlike other studies that used open‐ended formats (e.g., Roaldset et al. 2023). There is also a possibility that the respondents did not receive equal training for the use of the instrument, as studies with more critical feedback had no further details on what kind of training had been given on the use of the instrument (Faay et al. 2013; Roaldset et al. 2023). For example, a previous BVC study found a lack of training as a reason for the non‐success of the assessment (Yuniati et al. 2020). However, based on this review, the feasibility outcomes of the review of the instruments were limited, as only half of the feasibility was studied. Nevertheless, more feasibility studies are needed for these short‐term assessment instruments and management strategies in institutional settings among adolescents to assess which is suitable in each environment. In addition, feasibility studies are also needed to determine how to efficiently implement a valid instrument or concrete strategy as part of daily practices.

There is a lack of clarity about how the measurements were made in some of the selected studies. For example, three studies did not report how often the instrument was applied/used (Chu et al. 2012; Faay et al. 2013; Eisenstein et al. 2023). In our understanding, the reasons for not doing the assessments were possibly due to, for example, haste (Chu et al. 2012), lack of clarity on how to use the instruments, or lack of confidence (Yuniati et al. 2020). Therefore, it is difficult to meaningfully judge the outcomes of these instruments if they are not being used properly. There is a need to gain a better understanding of how these instruments are used and how they can be used regularly without the use of the instrument feeling like additional work from the employees' point of view (Koh et al. 2020). Therefore, more research is needed to clarify and simplify the use of these short‐term instruments, for example, by studying whether instrument users would benefit from digitalising and integrating the instruments into patient information systems (Ratwani 2017). The inability to demonstrate effectiveness in mitigating the risk of violence is a major reason why these methods are not adopted in practice, even though they have value in structuring judgements and informing personal formulations of needs and risks (Viljoen et al. 2018). Future research should prioritise links with risk formulation, formulation‐informed risk management, protective factors and detailed investigations into the practical utility of structured guidelines and instruments (Levin 2019).

While many of the reviewed studies emphasise psychometric properties, they underemphasise implementation issues critical to mental health nursing. Since nurses primarily encounter youths and spend the most time interacting with them compared to other professional groups, they are expected to manage and respond to the level of risk, highlighting a clear research bias (Delaney and Johnson 2014). They are typically responsible for administering these instruments; thus, understanding their usability, interpretability and alignment with nursing care models is vital (Simpson and McKenna 2014; Lantta et al. 2016). The studies selected for this review also indicate that scoring was conducted by clinical staff, the majority of whom were nurses. In addition, assessors were not blinded and have downsides regarding causal relations. Paradoxically, high‐risk youths may become low‐risk because those predicted as high‐risk in clinical situations are obligated to manage the risk. This risk paradox is widespread in prediction‐outcome research, where researchers use data from real‐time clinical measures (van Geloven et al. 2024).

There is a need for studies in which blind, non‐clinical assessors conduct assessments, utilising a prospective design that ensures outcomes remain unknown to the assessors while maintaining ethical standards and participant safety. While the use of non‐clinical assessors in research can help reduce certain biases, particularly those related to the ‘risk paradox’ where risk assessments influence subsequent management (van Geloven et al. 2024), it is essential to balance this with ethical safeguards. Ethical standards can be maintained by ensuring that non‐clinical assessors are thoroughly trained in the use of the instruments, including understanding behavioural cues, confidentiality obligations and limits of their role. Furthermore, their involvement should be strictly limited to observational and assessment tasks, with all clinical decisions and interventions remaining under the purview of qualified healthcare professionals. Moreover, it is crucial to involve specialised Mental Health Nursing Practitioners (MHNPs) in the assessment, formulation and management of violence risk. Their expertise, ranging from technical skills like recognising early warning signs to the ability to build therapeutic relationships based on trust and support, enhances multidisciplinary collaboration and ensures more comprehensive care within mental health teams (Simpson and McKenna 2014). Ultimately, violence risk assessments should remain the responsibility of trained clinical staff. Research involving non‐clinical assessors is a methodological tool to improve instrument validation, not a substitute for professional clinical judgement in practice.

In addition, the lack of youth engagement in violence risk assessment stands out clearly. The V‐RISK‐Y assessment was reported to be made without adolescents and/or adolescents' parents (Laake et al. 2025; Roaldset et al. 2023). Other studies did not clarify whether adolescents were part of the assessments in any way, for example, going through assessments together or scoring in the presence of adolescents. Adolescents could be part of assessments, for example, going through assessments together after a violent incident or scoring in the presence of the youth. There are suggestions from the National Institute for Health and Care Excellence for staff to encourage adolescents to recognise signs and triggers of possible violent outcomes. Shared information with the staff about the triggers and warning signs identified by the adolescents themselves using the service can reduce the risk of violence (National Institute for Health and Care Excellence [NICE] 2019). There are already conclusions made about the need for adult engagement in risk assessment and management (Higgins et al. 2016; Lantta et al. 2016), but there is still a lack of research on youth engagement. Engagement in violent risk assessment and management can provide adolescents with feelings of autonomy, control and belonging (Sinclair et al. 2019). However, there is a need for evidence‐based recommendations regarding the impact of youth engagement in short‐term risk assessment instruments and management strategies.

This review has a few important limitations. First, despite searching in various databases and bibliographies in all languages, we identified only nine studies meeting our inclusion criteria. All observations are based on limited information regarding the conclusions drawn. Second, we might have overlooked certain relevant studies related to short‐term risk management practices as they fall under categories such as ‘anxiety relief,’ nevertheless, our search terms mainly focused on risk management terminology. To address this limitation, we consulted our research team for recommendations on potential studies; however, we did not receive any additional insights, and all available information has been included in our findings. Third, violence forms such as sexual, domestic and family violence were excluded because this review focused on violence against others in institutional settings. However, adolescents encounter many other forms of violence, such as sexual violence (UNICEF 2024) in many other settings besides institutional settings. Fourth, protective factors play an important role in preventing violence. Research is needed on how the measurement of protective factors could be utilised in the assessment and management of short‐term (24 h) violence, as the studies now focus on follow‐up measurements in years, such as 3‐year follow‐ups (Finseth et al. 2023).

## 6 Conclusion

This review identified nine studies that evaluated six different violence risk assessment instruments; however, short‐term violence risk management methods were not identified. We found that instruments developed more specifically for adolescent populations (DASA‐YV and V‐RISK‐Y) may be more accurate compared to instruments developed for adult populations (DASA). It would be useful to determine whether these age‐specific instruments and methods should only be used with adolescents. In addition, more research is needed on these youth‐specific instruments in different inpatient environments in the adolescent population. Even though risk assessment instruments may be a starting point for risk management (Viljoen et al. 2018), management methods are not included sufficiently and clearly in the valid instruments. Risk management must be understood as a distinct and equally critical phase of violence prevention. Based on this review, it is important to ensure that short‐term risk assessment and management strategies are optimally used as a part of day‐to‐day work in institutional settings, such as generating instruments for EHR (Electronic Health Record) systems or building more structured guidelines between assessment and management decisions to fit in an institutional environment. Future research should not only evaluate the predictive validity of short‐term assessment instruments but also investigate the development and implementation of evidence‐based strategies for managing identified risks within institutional youth care settings. Concerning engagement, adolescents could be part of assessments and, most importantly, give information on violence risk management strategies for future research.

## 7 Relevance for Clinical Practice

This review may help to secure resources and commitment at the service level to more suitable instruments for adolescents, such as DASA‐YV, which has been tested in similar environments with comparable patient populations in youth acute psychiatric units. This review highlights the importance of selecting instruments validated in mental health nursing practice and designed to support clinical judgement and emphasising observable risk markers within a 24‐h window. The conclusion provided by this review is that there is a need to further engage youth in their violence risk assessment and management. Clinicians can involve youth in assessing their own risk for violence, for example, by going through assessments together after a violent incident or scoring in the presence of the youth.

## Author Contributions

L.V., M.B., Y.L., V.P.‐P., A.F. and T.L. contributed substantially to the conception of the work. L.V., M.B. and T.L. acquired the data. L.V., M.B., Y.L. and T.L. analysed the data. L.V., M.B. and T.L. interpreted the data. L.V. drafted the manuscript. L.V., M.B., Y.L., V.P.‐P., A.F. and T.L. critically reviewed the article for its important intellectual content. All authors approved and agreed with the final version of the manuscript.

## Conflicts of Interest

The authors declare no conflicts of interest.

## Data Availability

Data sharing not applicable to this article as no datasets were generated or analysed during the current study.
